# Plasma Endocan as a Predictor of Cardiovascular Event in Patients with End-Stage Renal Disease on Hemodialysis

**DOI:** 10.3390/jcm9124086

**Published:** 2020-12-18

**Authors:** Jin Sug Kim, Gang Jee Ko, Yang Gyun Kim, So Young Lee, Dong Young Lee, Kyung Hwan Jeong, Sang Ho Lee

**Affiliations:** 1 Division of Nephrology, Department of Internal Medicine, Kyung Hee University School of Medicine, Seoul 02453, Korea; jinsuk0902@naver.com (J.S.K.); apple8840@hanmail.net (Y.G.K.); 2 Division of Nephrology, Department of Internal Medicine, Korea University College of Medicine, Seoul 02841, Korea; lovesba@hanmail.net; 3 Division of Nephrology, Department of Internal Medicine, CHA Bundang Medical Center, CHA University, Seongnam 13496, Korea; ysy0119@cha.ac.kr; 4 Division of Nephrology, Department of Internal Medicine, Veterans Health Service Medical Center, Seoul 05368, Korea; biizz@hanmail.net

**Keywords:** endocan, hemodialysis, end-stage renal disease, biomarker

## Abstract

Endocan, a potential biomarker of endothelial dysfunction, is associated with increased cardiovascular risk. We investigated the utility of plasma endocan for predicting cardiovascular risk in end-stage renal disease (ESRD) patients undergoing hemodialysis. Of the 452 patients in the K-cohort, 354 with available plasma endocan levels were enrolled. The correlation between plasma endocan levels and the clinical characteristics of a study population were analyzed. We divided patients into two groups, according to plasma endocan levels, and investigated the predictive value of endocan for the occurrence of cardiovascular events. In a multiple linear regression analysis, plasma endocan levels were positively correlated with previous cardiovascular events and negatively correlated with body mass index, albumin, and triglyceride. Patients with higher plasma endocan levels experienced more frequent cardiovascular events than those with lower plasma endocan levels (12.9% in the lower group vs. 22.7% in the higher group, *p* = 0.016). Cox proportional hazard models showed that higher plasma endocan levels were an independent predictor of cardiovascular events in ESRD patients on hemodialysis ((hazard ration) HR 1.949, 95% (confidence interval) CI 1.144–3.319, *p* = 0.014). Our results suggest that plasma endocan level might be a useful biomarker for predicting cardiovascular events in ESRD patients on hemodialysis.

## 1. Introduction

Patients with end-stage renal disease (ESRD) on hemodialysis have an increased mortality rate compared to the general population. Cardiovascular disease is the most common cause of death, accounting for about 40% to 50% of mortalities in ESRD patients [1]. In addition to traditional cardiovascular risk factors, such as hypertension, diabetes, and dyslipidemia, additional risk factors, including volume overload and disturbance of calcium and phosphate metabolism, can contribute to increased cardiovascular risk in ESRD patients [1,2]. A predictive tool for assessing cardiovascular risk in patients with ESRD would be beneficial. However, limited data are available on biological markers capable of predicting cardiovascular outcomes in patients with ESRD.

Endocan, a circulating proteoglycan secreted from vascular endothelial cells, affects the regulation of cell adhesion, migration, proliferation, and neovascularization in the endothelium [3,4]. Several studies have demonstrated that endocan is involved in endothelial dysfunction and inflammation, and could be an independent risk factor for poor clinical outcomes in various disease conditions [5,6,7,8]. Our research group previously reported that endocan is a potential biomarker of disease progression in patients with IgA nephropathy [9] and microvascular inflammation in kidney transplant patients [10]. Recently, endocan has emerged as a promising biomarker of cardiovascular disease. One meta-analysis demonstrated that serum endocan levels were significantly increased in patients with cardiovascular disease and suggested that endocan is a risk factor for cardiovascular disease [11]. Previous studies reported that higher endocan levels were associated with adverse cardiovascular outcomes in patients with various disease conditions, such as acute myocardial infarction, hypertension, and chronic kidney disease (CKD) [12,13,14]. However, to our knowledge, no study has been conducted to investigate the role of endocan in predicting cardiovascular risk in ESRD patients on hemodialysis.

In this study, we measured the levels of plasma endocan and analyzed its association with clinical characteristics in ESRD patients on hemodialysis. We also investigated the predictive value of endocan for cardiovascular outcomes based on more than two years of follow-up observations.

## 2. Materials and Methods

### 2.1. Study Population and Design

The study population was derived from the K-cohort, a multicenter, Internet-based, prospective cohort of patients with ESRD on hemodialysis in South Korea. The K-cohort was developed to evaluate the mortality and morbidity of ESRD patients, starting from 2016 (CRIS no. KCT0003281). ESRD patients from six hospitals, who were over 18 years of age and on hemodialysis, were included in the K-cohort if they were undergoing hemodialysis three times a week for over three months. Inclusion/exclusion criteria and detailed cohort design have been described previously [15,16]. Of the 452 patients who were enrolled as part of the K-cohort from June 2016 to August 2019, 354 with available plasma endocan data were included in this study. The study population was divided into two groups: lower endocan and higher endocan groups. We compared baseline characteristics and clinical outcomes between the two groups, and analyzed the association of plasma endocan levels with clinical parameters. We also investigated the predictive value of endocan for adverse cardiovascular outcomes.

The study was conducted in accordance with the Declaration of Helsinki, and the institutional review board of each participating center approved the study protocol (KHNMC IRB No. 2016-04-039). Written consent was obtained from all participants involved in this study before enrollment.

### 2.2. Variables and Study Outcome

We comprehensively collected baseline variables, including age, sex, body mass index (BMI), prior medical history, comorbid conditions, dialysis information, laboratory data, and medications. Blood samples for laboratory data were collected in the fasting state, just before the start of hemodialysis in a midweek session. The primary study outcome was a cardiovascular event, which was defined as the development of at least one of the following conditions: acute coronary syndrome (ACS), stable angina requiring percutaneous coronary intervention (PCI) or coronary artery bypass grafting (CABG), heart failure, ventricular arrhythmia, cardiac arrest, and sudden death. Disease-free survival was measured from the date of enrollment to the first occurrence of composite cardiovascular outcomes. All patients were monitored for the development of study outcomes until March 2020.

### 2.3. Laboratory Analysis

Routine biochemical laboratory parameters were measured using standard methods. We collected plasma samples using tubes treated with ethylenediaminetetraacetic acid (EDTA) on the day of enrolment in the study. After centrifugation for 15 min at 1000× *g* at room temperature, samples were stored at −80 °C until analysis. We measured endocan levels using an enzyme-linked immunosorbent assay (ELISA) using Magnetic Luminex^®^ Screening Assay multiplex kits (R&D Systems, Minneapolis, MN, USA), as described previously [9,10].

### 2.4. Statistical Analysis

Continuous variables are described as means ± standard deviations (SDs) and were analyzed using Student’s t-test or the Mann–Whitney U test. Categorical variables are presented as frequencies and percentages. Categorical data were analyzed using the chi-square test or Fisher’s exact test. Linear regression analyses were performed to assess the correlations between baseline clinical characteristics. The R package MaxStat (R Foundation for Statistical Computing, Vienna, Austria) was used to determine the optimal endocan cut-off value. In an analysis of all patients, a cut-off value of 1023.52 pg/mL was found to have the highest log-rank statistic of any cut-off value. Kaplan–Meier estimates of time to event endpoints were calculated. Multivariate Cox regression analyses were performed with all univariate associations of *p* < 0.1 to identify a predictive model for cardiovascular event risk. The results are presented as hazard ratios (HRs) and ±95% confidence intervals (CIs), and statistical significance is indicated. Statistical analyses were conducted using SPSS software version 19.0 (SPSS Inc., Chicago, IL, USA) and R software version R 4.0.2 (R Foundation for Statistical Computing, Vienna, Austria). A *p*-value < 0.05 was considered to indicate statistical significance.

## 3. Results

### 3.1. Baseline Clinical Characteristics

A total of 354 adult patients with ESRD who were undergoing hemodialysis at six hospitals were enrolled in this study. Patients were divided into two groups, according to the optimal cut-off value of plasma endocan levels, which were obtained using the aforementioned method: a lower endocan group (endocan 1023.52 pg/mL, *n* = 178) and a higher endocan group (endocan ≥ 1023.52 pg/mL, *n* = 176). Baseline characteristics of the study population, according to plasma endocan levels, are summarized in Table 1. Patients in the higher endocan group had a lower BMI (*p* < 0.001) and a lower prevalence of diabetes mellitus (*p* = 0.001). Albumin and glucose levels were significantly lower in the higher endocan group than in the lower endocan group (*p* = 0.032 and *p* = 0.012, respectively). Patients with higher endocan levels had longer hemodialysis duration (*p* < 0.001) and higher single-pool Kt/V (*p* = 0.013) than patients with lower endocan levels. There was a significant difference in lipid profiles and medications between the two groups.

### 3.2. Associations between Plasma Endocan Level and Baseline Characteristics

Table 2 shows associations between plasma endocan level and various clinical characteristics in patients with ESRD on hemodialysis. In univariate linear regression analyses, hemodialysis duration, single-pool Kt/V, and HDL-cholesterol were positively associated with plasma endocan level. Plasma endocan level was inversely associated with BMI, diabetes mellitus, albumin, glucose, total cholesterol, and triglyceride. Variables with *p*-values < 0.10 in the univariate analysis were included for multivariate analysis. In multivariate linear regression analysis, endocan level was positively associated with a previous cardiovascular event (*β* = 0.106; *p* = 0.039) and negatively associated with BMI (*β* = −0.170; *p* = 0.005), albumin (*β* = −0.118; *p* = 0.026), and triglyceride (*β* = −0.155; *p* = 0.025).

### 3.3. Study Outcome in Accordance with Plasma Endocan Level

**Figure 1:**
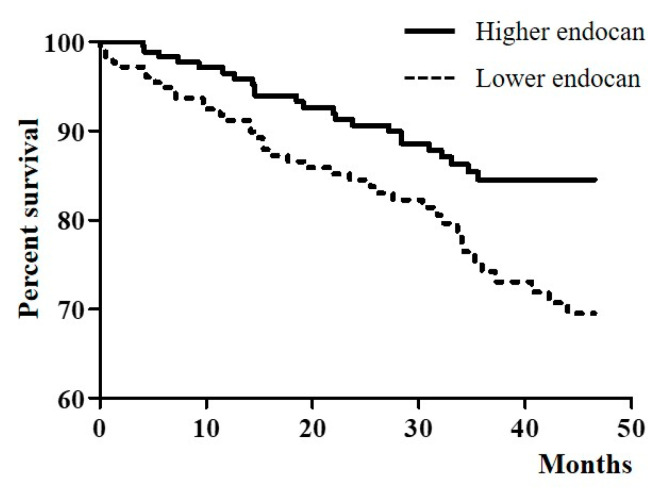
Cardiovascular event-free survival according to plasma endocan level.

The prevalence of cardiovascular events, according to plasma endocan levels, is shown in Table 3. The mean follow-up duration was 34.56 months. During the follow-up period, 63 (17.8%) patients experienced cardiovascular events. Of the 63 patients, 19 (5.4%) had ACS, 11 (3.1%) had stable angina, requiring PCI or CABG, 7 (2.0%) had heart failure, 3 (0.8%) had ventricular arrhythmia, and 23 (6.5%) had cardiac death. Patients with higher plasma endocan levels frequently experienced cardiovascular events (12.9% in the lower group vs. 22.7% in the higher group, *p* = 0.016). Figure 1 shows cardiovascular event-free survival, according to plasma endocan levels. The log-rank test identified a significant association between the endocan level and cardiovascular event-free survival (*p* = 0.006). To analyze the cause of death, we also observed the prevalence of non-cardiac death. Thirty-seven (10.5%) patients experienced non-cardiac death and the most common cause of non-cardiac death was infection. Patients with higher plasma endocan levels showed a more frequent prevalence of non-cardiac death (6.2% in the lower group vs. 14.8% in the higher group, *p* = 0.008) (Table S1).

Univariate and multivariate Cox regression analyses were conducted to identify risk factors associated with cardiovascular events in ESRD patients on hemodialysis (Table 4). In univariate Cox regression analysis, diabetes mellitus (HR 2.287, 95% CI 1.1311–3.990, *p* = 0.004), previous cardiovascular events (HR 2.308, 95% CI, 1.392–3.825, *p* = 0.001), glucose (HR 1.005, 95% CI, 1.002–1.008, *p* = 0.004), use of angiotensin II receptor blockers or angiotensin-converting enzyme inhibitor (HR 1.746, 95% CI 1.011–3.016, *p* = 0.046), use of calcium channel blocker (HR 1.864, 95% CI 1.044–3.329, *p* = 0.035), use of statin (HR 1.701, 95% CI 1.030–2.811, *p* = 0.038), use of antidiabetic drugs (HR 2.018, 95% CI 1.218–3.345, *p* = 0.006), and higher plasma endocan levels (HR 1.994, 95% CI, 1.193–3.334, *p* = 0.008) showed a significant association with the development of cardiovascular events in ESRD patients on hemodialysis. After adjustment for variables with a *p*-value < 0.10 in the univariate analysis, previous cardiovascular events (HR 1.976, 95% CI, 1.139–3.430, *p* = 0.015) and higher plasma endocan levels (HR 1.949, 95% CI 1.144–3.319, *p* = 0.014) were independent factors associated with the development of cardiovascular events in patients with ESRD on hemodialysis.

## 4. Discussion

In this study, we measured plasma endocan levels and investigated its correlation with clinical characteristics in ESRD patients on hemodialysis, using a prospective multicenter cohort. We also determined the predictive value of plasma endocan levels on the development of cardiovascular events in ESRD patients on hemodialysis. Our principal findings are that, in patients with ESRD undergoing hemodialysis, (1) plasma endocan levels were positively correlated with previous cardiovascular events and negatively correlated with BMI, albumin, and triglyceride; (2) patients with higher plasma endocan levels experienced cardiovascular events more frequently than those with lower plasma endocan levels; (3) higher plasma endocan level was an independent predictor of cardiovascular events, even after adjusting for various clinical parameters.

Endocan has been reported to be associated with the occurrence and clinical outcomes of various disease conditions, such as hypertension, sepsis, and malignancy [17,18,19,20]. The association between endocan and kidney diseases has also been studied. Gunay et al. [21] reported that serum endocan levels were significantly increased in patients with acute kidney injury (AKI). Other researchers have suggested serum endocan as a potential biomarker for differentiating causes of intrinsic AKI [22]. Yilmaz et al. [6] demonstrated that plasma endocan levels are correlated with decreasing estimated glomerular filtration rate, and influence cardiovascular events, and all-cause mortality in non-dialysis patients with CKD.

The clinical relevance of endocan has also been analyzed in ESRD patients who needed renal replacement therapy. In kidney transplant patients, serum endocan levels were significantly correlated with chronic renal allograft injury [23]. In our previous study, plasma and urinary endocan levels were significantly increased in kidney transplant patients with acute antibody-mediated rejection, and patients with high endocan levels showed worse renal survival [10]. In ESRD patients undergoing peritoneal dialysis, serum endocan levels have a predictive value for decreasing urine volume and showed a significant correlation with clinical characteristics and cardiovascular events [24,25]. However, only a few studies have evaluated endocan in hemodialysis patients [26,27], and the studies were conducted with a relatively small sample size, and did not analyze the association between clinical outcomes and endocan levels.

In the present study, we demonstrated the clinical relevance of endocan in patients with ESRD on hemodialysis. We found that endocan was inversely correlated with BMI, albumin, and triglyceride levels. Given that these factors are widely accepted as showing nutritional status in hemodialysis patients, endocan might be a potential indicator of nutritional status in patients with ESRD on hemodialysis. To determine the association between endocan level and nutritional status, we calculated the geriatric nutritional risk index (GNRI) [28] and performed further analyses. GNRI was significantly lower in the higher endocan group than in the lower endocan group (91.89 ± 7.42 in the lower endocan group and 87.17 ± 7.13 in the higher endocan group, *p* < 0.001). Endocan levels showed a negative correlation with GNRI (r = −0.294, *p* < 0.001). Similar to our findings, Poon et al. [25] reported that serum endocan levels correlated with the nutritional status of ESRD patients on peritoneal dialysis. In their study, serum endocan levels were inversely correlated with serum albumin levels, and patients with higher endocan levels had a lower subjective global assessment scale and a higher malnutrition-inflammation score. Further studies are needed to establish the mechanism that underlies the relationship between nutritional status and endocan levels.

In our study, the lower endocan group showed significantly lower single-pool Kt/V compared with the higher endocan group (1.53 ± 0.29 in the lower endocan group and 1.61 ± 0.30 in the higher endocan group, *p* = 0.013). Although no further analysis was conducted on this result, the following supposition can be made: given that patients in the lower endocan group have shorter hemodialysis (HD) duration than those in the higher endocan group, it is assumed that patients in the lower endocan group have a higher residual renal function. Previous studies reported that endocan levels showed a negative association with residual renal function [24,25]. The result of adding residual renal function to assess dialysis adequacy may differ from our prior result. However, we could not measure residual renal function due to the limitation of cohort design. To overcome this limitation, we are planning an additional cohort study with the modification of cohort design.

The results of the present study suggest that plasma endocan levels can be used as an independent cardiovascular risk factor, consistent with previous studies. A recent meta-analysis showed that endocan levels were significantly increased in patients with cardiovascular disease [11]. In another study, plasma endocan levels were associated with cardiovascular events and all-cause mortality [29]. Pawlak et al. [14] showed that plasma endocan levels were significantly increased in patients with CKD, and independently associated with soluble intercellular adhesion molecule-1 and soluble vascular cell adhesion molecule-1, which affect the prevalence of cardiovascular disease in these patients. Our findings provide important evidence of the utility of endocan as a predictive biomarker of cardiovascular risk in patients with ESRD on hemodialysis.

Our study had some potential limitations. Firstly, the plasma endocan level was measured only once, which may have resulted in the incorrect classification of patients. To overcome this limitation, we intend to establish an additional independent cohort to validate our findings. Secondly, the cut-off value of higher and lower serum endocan was chosen following the appropriate calculation; however, further studies with large sample sizes are needed to ascertain the reliability of the cut-off value. Thirdly, since the patients enrolled in this study were predominantly Korean, the results should be generalized with caution, and further studies are needed to assess the clinical relevance of endocan levels in different populations. However, our study is significant because it is the first study to demonstrate a significant association between plasma endocan levels and cardiovascular events in ESRD patients on hemodialysis, using a relatively large number of patients from a prospective multicenter cohort.

In conclusion, ESRD patients on hemodialysis with higher plasma endocan levels showed more frequent cardiovascular events than those with lower plasma endocan levels. Higher plasma endocan level was an independent predictor of cardiovascular events in patients with ESRD on hemodialysis. Plasma endocan levels could be a useful predictive marker of the risk of cardiovascular events in patients with ESRD on hemodialysis. Further studies involving multiethnic and/or larger numbers of participants are needed to ascertain the underlying mechanism and validate our findings.

## Figures and Tables

**Table 1:** Baseline characteristics according to plasma endocan level.

	Lower Endocan Group (*n* = 178)	Higher Endocan Group (*n* = 176)	*p*
	<1023.52 pg/mL	≥1023.52 pg/mL	
Age (year)	60.84 ± 13.53	63.45 ± 11.82	0.054
Sex (Male, %)	126 (70.8%)	109 (61.9%)	0.078
Body mass index (kg/m^2^)	24.35 ± 4.20	21.78 ± 3.18	<0.001
Hypertension (*n*, %)	154 (86.5%)	149 (84.7%)	0.619
Diabetes mellitus (*n*, %)	115 (64.6%)	83 (47.2%)	0.001
Previous cardiovascular event (*n*, %)	67 (37.6%)	82 (46.6%)	0.088
Cause of ESRD (*n*, %)			0.024
Diabetes mellitus	97 (54.5%)	69 (39.2%)	
Hypertension	25 (14.0%)	47 (26.7%)	
Glomerulonephritis	20 (11.2%)	22 (12.5%)	
ADPKD	11 (6.2%)	8 (4.5%)	
Others	21 (11.8%)	27 (15.3%)	
Unknown	4 (2.2%)	3 (1.7%)	
HD duration (month)	46.62 ± 53.01	72.77 ± 76.65	<0.001
Average ultrafiltration (L)	2.31 ± 1.06	2.17 ± 1.05	0.222
Single-pool Kt/V	1.53 ± 0.29	1.61 ± 0.30	0.013
Pre-dialysis SBP (mmHg)	141.69 ± 19.58	145.53 ± 20.55	0.073
Pre-dialysis DBP (mmHg)	72.14 ± 12.43	73.09 ± 13.55	0.492
Laboratory findings			
Hemoglobin (g/dL)	10.52 ± 1.24	10.34 ± 1.21	0.187
Albumin (g/dL)	3.85 ± 0.31	3.78 ± 0.34	0.032
Creatinine (mg/dL)	9.46 ± 2.87	9.31 ± 2.79	0.615
Ca × P	41.37 ± 12.05	40.20 ± 12.98	0.379
Glucose (mg/dL)	161.91 ± 66.20	145.19 ± 57.41	0.012
Total cholesterol (mg/dL)	141.01 ± 31.62	130.48 ± 27.42	0.001
Triglyceride (mg/dL)	147.46 ± 90.70	93.30 ± 46.42	<0.001
LDL cholesterol (mg/dL)	80.48 ± 26.70	73.04 ± 24.45	0.007
HDL cholesterol (mg/dL)	42.46 ± 13.17	47.13 ± 12.27	0.001
hs-CRP (mg/dL)	3.91 ± 8.29	3.88 ± 7.89	0.973
Medications (*n*, %)			
ARB or ACEi	102 (57.3%)	108 (61.4%)	0.437
CCB	104 (58.45)	113 (64.2%)	0.264
Beta-blocker	60 (33.7%)	90 (51.1%0	0.001
Statin	83 (46.6%)	83 (47.2%)	0.920
Anti-platelet drugs	124 (69.7%)	128 (72.7%)	0.524
Anti-diabetic drugs	95 (54.3%)	60 (34.3%)	<0.001

ESRD, end-stage renal disease; ADPKD, autosomal dominant polycystic kidney disease; HD, hemodialysis; SBP, systolic blood pressure; DBP, diastolic blood pressure; LDL, low-density lipoprotein; HDL, high-density lipoprotein; hs-CRP, high-sensitivity C-reactive protein; ARB, angiotensin II receptor blocker; ACEi, angiotensin-converting enzyme inhibitor; CCB, calcium channel blocker.

**Table 2:** Association between plasma endocan level and various clinical characteristics.

	Simple Regression				Multiple Regression			
	B	SE	Beta	*p*	B	SE	Beta	*p*
Age (years)	3.262	2.602	0.067	0.211				
Male sex	−68.797	70.252	−0.052	0.328				
Body mass index (kg/m^2^)	−44.83	8.097	−0.283	<0.001	−26.871	9.535	−0.170	0.005
Hypertension	47.018	94.602	0.026	0.619				
Diabetes mellitus	−217.213	55.928	−0.173	0.001	−45.131	108.377	−0.036	0.680
Previous cardiovascular event	128.925	66.96	0.102	0.055	134.133	64.726	0.106	0.039
HD duration (month)	1.591	0.491	0.171	0.001	0.644	0.511	0.069	0.209
Average ultrafiltration (L)	−17.422	31.454	−0.03	0.58				
Single-pool Kt/V	243.421	111.197	0.116	0.029	−76.845	124.517	−0.036	0.538
Pre-dialysis SBP (mmHg)	3.059	1.645	0.099	0.064	2.962	1.755	0.095	0.092
Pre-dialysis DBP (mmHg)	4.726	2.548	0.098	0.065	3.224	2.768	0.067	0.245
Laboratory findings								
Hemoglobin (g/dL)	−54.434	26.931	−0.107	0.044	−22.458	27.032	−0.044	0.407
Albumin (g/dL)	−264.965	100.896	−0.139	0.009	−225.804	101.063	−0.118	0.026
Creatinine (mg/dL)	−6.573	11.764	−0.03	0.577				
Ca × P	−3.18	2.653	−0.064	0.231				
Glucose (mg/dL)	−1.593	0.526	−0.159	0.003	−0.907	0.606	−0.091	0.136
Total cholesterol (mg/dL)	−2.308	1.101	−0.111	0.037	−0.497	2.178	−0.024	0.820
Triglyceride (mg/dL)	−2.554	0.41	−0.315	<0.001	−1.253	0.555	−0.155	0.025
LDL cholesterol (mg/dL)	−2.244	1.282	−0.093	0.081	0.214	2.233	0.009	0.923
HDL cholesterol (mg/dL)	8.838	2.531	0.183	0.001	3.447	3.492	0.070	0.324
hs-CRP (mg/dL)	1.676	4.115	0.022	0.684				
Medications								
ARB or ACEi	98.081	67.448	0.077	0.147				
CCB	111.872	69.029	0.086	0.106				
Beta-blocker	91.817	67.072	0.073	0.172				
Statin	−21.543	66.583	−0.017	0.746				
Anti-platelet drugs	−23.996	73.365	−0.327	0.744				
Anti-diabetic drugs	−201.941	66.601	−0.160	0.003	−30.054	104.717	−0.024	0.774

HD, hemodialysis; SBP, systolic blood pressure; DBP, diastolic blood pressure; LDL, low-density lipoprotein; HDL, high-density lipoprotein; hs-CRP, high-sensitivity C-reactive protein; ARB, angiotensin II receptor blocker; ACEi, angiotensin-converting enzyme inhibitor; CCB, calcium channel blocker.

**Table 3:** Clinical outcomes according to plasma endocan level.

	Overall	Lower Endocan Group	Higher Endocan Group	*p*
	(*n* = 354)	(*n* = 178)	(*n* = 176)	
Follow-up duration (month)	34.56 ± 13.86	35.99 ± 13.69	33.10 ± 13.92	0.051
Cardiovascular events	63 (17.8%)	23 (12.9%)	40 (22.7%)	0.016
Acute coronary syndrome	19 (5.4%)	7 (3.9%)	12 (6.8%)	
Stable angina with PCI or CABG	11 (3.1%)	4 (2.2%)	7 (4.0%)	
Heart failure	7 (2.0%)	2 (1.1%)	5 (2.8%)	
Ventricular arrhythmia	3 (0.8%)	1 (0.6%)	2 (1.1%)	
Cardiac death or sudden death	23 (6.5%)	9 (5.1%)	14 (8.0%)	

PCI, percutaneous coronary intervention; CABG, coronary artery bypass graft.

**Table 4:** Predictors of cardiovascular events in univariate and multivariate Cox regression analyses.

Variable	Univariate Analysis	*p*	Multivariate Analysis	*p*
	HR (95% CI)		HR (95% CI)	
Age (year)	1.017 (0.998–1.036)	0.075	1.007 (0.986–1.030)	0.512
Male sex	0.774 (0.465–1.286)	0.322		
Body mass index (kg/m^2^)	0.976 (0.916–1.040)	0.453		
Hypertension	2.090 (0.838–5.211)	0.114		
Diabetes mellitus	2.287 (1.311–3.990)	0.004	1.554 (0.656–3.685)	0.316
Previous cardiovascular event	2.308 (1.392–3.825)	0.001	1.976 (1.139–3.430)	0.015
HD duration (month)	0.998 (0.994–1.002)	0.409		
Average ultrafiltration (L)	0.987 (0.781–1.247)	0.987		
Single-pool Kt/V	0.639 (0.278–1.468)	0.291		
Pre-dialysis SBP (mmHg)	1.005 (0.993–1.018)	0.398		
Pre-dialysis DBP (mmHg)	0.991 (0.971–1.012)	0.395		
Laboratory findings				
Hemoglobin (g/dL)	0.870 (0.719–1.052)	0.15		
Albumin (g/dL)	0.621 (0.288–1.343)	0.226		
Creatinine (mg/dL)	0.929 (0.848–1.019)	0.118		
Ca × P	0.996 (0.975–1.017)	0.716		
Glucose (mg/dL)	1.005 (1.002–1.008)	0.004	1.002 (0.998–1.006)	0.286
Total cholesterol (mg/dL)	0.994 (0.985–1.003)	0.164		
Triglyceride (mg/dL)	1.001 (0.997–1.004)	0.736		
LDL cholesterol (mg/dL)	0.998 (0.988–1.008)	0.962		
HDL cholesterol (mg/dL)	0.992 (0.973–1.012)	0.425		
hs-CRP (mg/dL)	0.997 (0.965–1.031)	0.882		
Medications				
ARB or ACEi	1.746 (1.011–3.016)	0.046	1.292 (0.712–2.345)	0.399
CCB	1.864 (1.044–3.329)	0.035	1.724 (0.911–3.262)	0.095
Beta-blocker	1.038 (0.630–1.709)	0.885		
Statin	1.701 (1.030–2.811)	0.038	1.295 (0.766–2.191)	0.334
Antiplatelet drugs	1.557 (0.846–2.866)	0.155		
Antidiabetic drugs	2.018 (1.218–3.345)	0.006	1.296 (0.588–2.858)	0.520
Endocan levels				
Lower	1		1	
Higher	2.027 (1.213–3.389)	0.007	1.949 (1.144–3.319)	0.014

HD, hemodialysis; SBP, systolic blood pressure; DBP, diastolic blood pressure; LDL, low-density lipoprotein; HDL, high-density lipoprotein; hs-CRP, high-sensitivity C-reactive protein; ARB, angiotensin II receptor blocker; ACEi, angiotensin-converting enzyme inhibitor; CCB, calcium channel blocker.

## Supplementary Materials

The following are available online at https://www.mdpi.com/2077-0383/9/12/4086/s1, Table S1: Non-cardiac death according to plasma endocan level.
