# Supplementary material for: Plasma Endocan as a Predictor of Cardiovascular Event in Patients with End-Stage Renal Disease on Hemodialysis
Source: J Clin Med. 2020 Dec 18;9(12):4086. doi: 10.3390/jcm9124086 (PMC7766985; doi:10.3390/jcm9124086)
Supplement: Supplementary file 1 [file jcm-09-04086-s001.pdf]

Supplementary file

Table 1. Non-cardiac death according to plasma endocan level.

	**Overall** (n = 354)	**Lower endocan group** (n = 178)	**Higher endocan group** (n = 176)	p
**Non-cardiac death**	37 (10.5%)	11 (6.2%)	26 (14.8%)	0.008
Infection	16 (4.5%)	4 (2.2%)	12 (6.8%)	
Cerbrovascular diseases	5 (1.4%)	2 (1.1%)	3 (1.7%)	
Others	6 (1.7%)	2 (1.1%)	4 (2.3%)	
Unknown	10 (2.8%)	3 (1.7%)	7 (4.0%)	
